# CRABP1 Enhances the Proliferation of the Dermal Papilla Cells of Hu Sheep through the Wnt/β-catenin Pathway

**DOI:** 10.3390/genes15101291

**Published:** 2024-09-30

**Authors:** Zahid Hussain, Tingyan Hu, Yuan Gou, Mingliang He, Xiaoyang Lv, Shanhe Wang, Wei Sun

**Affiliations:** 1 College of Animal Science and Technology, Yangzhou University, Yangzhou 225009, China;zahidchem224@gmail.com (Z.H.); hty980105@163.com (T.H.); gou1030923@163.com (Y.G.); 13013706620@163.com (M.H.); dx120170085@yzu.edu.cn (X.L.); 007121@yzu.edu.cn (S.W.); 2 Joint International Research Laboratory of Agriculture and Agri-Product Safety, Ministry of Education of China, Yangzhou University, Yangzhou 225009, China; 3 International Joint Research Laboratory in Universities of Jiangsu Province of China for Domestic Animal Germplasm Resources and Genetic Improvement, Yangzhou University, Yangzhou 225009, China

**Keywords:** CRABP1, sheep, dermal papilla cells, proliferation, Wnt/β-catenin

## Abstract

Background: The homologous proteins identified as cellular retinoic acid-binding proteins I and II (*CRABP-I* and *CRABP-II*) belong to a subset of intracellular proteins characterized by their robust affinity for retinoic acid, which plays an indispensable role in the development of hair follicle, including differentiation, proliferation, and apoptosis in keratinocytes. Previous research on Hu sheep hair follicles revealed the specific expression *CRABP1* in dermal papilla cells (DPCs), suggesting that *CRABP1* has a potential role in regulating the DPC population. Therefore, the main purpose of this study is to expose the performance of the *CRABP1* genes in the development and proliferation of DPCs. Methods: Initially, overexpression and inhibition of CRABP1 in the DPCs were conducted through overexpression vector and siRNA. CCK-8, EDU, and RT-PCR cell cycle assays and immunostaining were performed to evaluate the proliferation and cell cycle of dermal papilla cells (DPCs). Although, the influence of CRABP1 upon β-catenin in dermal papilla cells (DPCs) was found using immunofluorescence labeling. Finally, RT-PCR was conducted to assess the impact of CRABP1 on the expression levels of CTNNB1, TCF4, and LEF1 in DPCs involved in the Wnt/β-catenin signaling pathway. Results: The results showed that CRABP1 overexpression promotes the growth rates of DPCs and significantly enhances the proportion of S-phase cells compared with the control group (*p* < 0.05). The results were the opposite when *CRABP1* was a knockdown. In contrast, there was a significant decline in the mRNA expression levels of *CTNNβ1*, *LEF1* (*p* < 0.05), and *TCF4* (*p* < 0.01) by *CRABP1* knockdown. Conclusions: This study found that *CRABP1* influences the expression of important genes within the Wnt/β-catenin signaling pathway and promotes DPC proliferation. This investigation provides a theoretical framework to explain the mechanisms that control hair follicle morphogenesis and development.

## 1. Introduction

Wool is an essential component in the global textile industry, serving as the primary material for woolen products [1]. Maximizing profitability in the wool industry typically depends on the production of high-quality wool [2,3]. The two main cell types found in a hair follicle are dermal cells and epithelial cells. The hair shaft, accompanying layer, hair matrix, inner and outer root sheaths, and hair shaft comprise the epithelial portion of the follicle. The follicular stroma, which consists of the connective tissue sheath and derma papilla, surrounds this epithelial structure [4]. The secretion of a large number of growth factors by derma papilla cells (DPCs) regulates the development and growth of hair follicles. These growth factors facilitate the dermal-epidermal signal transduction, cell proliferation, and development within hair follicle cells [5]. Kwack and his colleagues have demonstrated that exosomes produced from cultured dermal papilla cells (DPCs) enhance the regeneration of human hair follicles, facilitate the transition of mouse hair from the telogen to the anagen phase, and promote the growth of outer root sheath cells and dermal papilla. This indicates that DPCs stimulate hair growth by using exosomes to regulate different cells within the hair follicle [6]. Moreover, the presence of dermal papillae has been shown to enhance the formation of entire hair follicles and stimulate the growth of hairs [7]. Therefore, studying the mechanism governing DPC proliferation affects the formation of hair follicles.

Previous research on Hu sheep hair follicles of Hu sheep revealed that dermal papilla cells (DPCs)express CRABP1 specifically. Cellular retinoic acid-binding proteins I and II (CRABPI and CRABPII), which are homologous proteins, belong to a group of intracellular proteins that demonstrate a high degree of affinity for binding retinoic acid [8]. All vertebrates have CRABPs, which are substantially conserved across species [9]. The expression of CRABPI is almost universal in adults, whereas both CRABP1 and CRABPII typically do not coexist in the same cells but are broadly expressed in the embryo [10]. There is an intriguing relationship between hair follicles and CRABP1 genes, which facilitate retinoic acid transport and interaction with nuclear receptors and are essential for regulating cellular processes such as differentiation and proliferation of DPCs [11]. CRABP1’s expression and activity in DPCs can influence hair characteristics such as follicle size, shape, and growth cycle [12]. DPCs are essential for the development and maintenance of hair follicles during their growth cycle [13]. In addition, CRABP1 plays an essential role in the basic signaling pathways between dermal papilla and epithelial cells, which are necessary for the development of follicles, the production of hair shafts, and the cycling process [14,15]. These pathways regulate a number of biological activities, including cell division and proliferation [16,17]. Understanding the relationship between CRABP1 and Hu sheep hair follicles could provide insight into the molecular processes that underlie the breed’s ability to produce wool.

Many studies have demonstrated that a number of molecular signaling pathways, including Wnt/β-catenin, play a key role in regulating DPCs [18,19]. A classic pathway, Wnt/β-catenin signaling facilitates the growth of hair as well as triggers downstream targets that enhance the proliferation of DPC [20,21]. Meanwhile, maintaining the stem cell properties of dermal papilla cells (DPCs) requires the initiation of the Wnt/β-catenin signaling pathway. These characteristics are essential for the continuous regeneration and cycling of hair follicles in sheep [22,23]. The Wnt/β-catenin pathway interacts with numerous growth factors and cytokines to promote the growth of DPCs [24]. Research indicates that the classic Wnt/β-catenin pathway is crucial for the activation of hair follicle formation, morphogenesis, and the regeneration of hair follicle DPCs, as well as promoting hair stem cell growth [25,26]. However, the interaction between the Wnt/β-catenin signaling pathways and CRABP1 remains unclear. This study aimed to validate the influence of CRABP1 on the proliferation of dermal papilla cells (DPCs) in Hu sheep and the regulation of the Wnt/β-catenin signaling pathway. The findings of this study may elucidate the mechanisms that promote hair follicle development.

## 2. Materials and Methods

### 2.1. Ethics Statement

All protocols for experiments using animals in this study have been carefully designed in accordance with the “Jiangsu Province Laboratory Animal Management Measures”. All protocols have been approved by Yangzhou University’s Animal Ethics Committee (No. NSFC2020NFY-1).

### 2.2. Animals, Cell Isolation, and Culture of DPCs

The cells have been extracted from Hu sheep sourced from a sheep farm in Suzhou, Jiangsu, China. The collection procedure for DPCs of Hu sheep included incising the skin tissue with ophthalmic scissors and subsequently extracting individual hair follicles from tiny portions of the dermal tissue. Subsequently, we carefully removed the dermal papilla from the hair follicle by grasping the larger portion with tweezers. Finally, the expanded portions of the hair follicles were planted into a 12-well plate to promote the growth of DPCs. The cell culture medium used for Hu sheep DPCs was Dulbecco’s Modified Eagle Medium F12 (DMEMF12) (HyClone, Logan, UT, USA). DPCs were cultured at 37 °C with 5% CO_2_, 10% fetal bovine serum, and 1% penicillin–streptomycin (Gibco, New York, NY, USA) as a supplement.

### 2.3. The Extraction of Total Cellular RNA, cDNA Synthesis, and RT—PCR

Total cellular RNA extraction was performed with Trizol Universal Reagent (Tiangen, Beijing, China) in accordance with the instructions provided by the manufacturer. After transfection, the DPCs in six-well plates were extracted when the cells achieved 80–90% confluence. The RNA was then kept at −80 °C for further experiments. A one-step reverse transcription kit from Tiangen (Beijing, China) was employed to generate cDNA from RNA. To evaluate the levels of related gene expression in cells and tissues, a 2×TSINGKE^®^ Master qPCR mix (Tsingke, Nanjing, China) was used. The gene used as a reference was GAPDH. Each sample underwent repeated testing three times; the relative expression was calculated using the2-delta delta CT method. Primer sequences for relevant genes were generated using Premier Primer 5.0, which was developed by Premier Biosoft International in Palo Alto, CA, USA. The primers were synthesized by Tsingke in Nanjing, China, as presented in Table 1.

### 2.4. CRABP1 over Expression Vector Construction

The predicted mRNA sequence for the sheep CRABP1 gene’s CDS full-length sequence (742 bp, Gene Bank Accession: XM_060401411.1) was made available by NCBI. Primer Premier 5.0 (Premier Biosoft International, Alto, CA, USA) was applied to design PCR amplification primers. The primer sequence is as follows:

CRABP1 F:ctagcgtttaaacttaagcttATGCCCAACTTCGCCGGC.

CRABP1 R:tgctggatatctgcagaattcTCATTCCCGAACATAAATCCTCG.

The full-length amplified CDS of CRABP1 is represented by the uppercase letters. The linearized vector pcDNA3.1’s homologous recombination terminal sequence is represented by the lowercase letters. The restriction enzyme digestion sites for EcoR1 are gaattc and aagctt for HindIII. The complete coding sequence (CDS) of CRABP1 was amplified using Prime STAR Max DNA polymerase (Takara, Beijing, China), and the results of the PCR were evaluated with 1% agarose gel electrophoresis. The SanPrep Column PCR Product Purification Kit was used to recover the PCR product. The concentration of DNA was carried out using a micro-ultraviolet spectrophotometer (Life Real, Hangzhou, China). The pcDNA3.1 (+) plasmid was cut using the restriction enzymes HindIII and EcoR1, and 1% agarose gel electrophoresis was used to identify the enzyme’s digestion products. The products of digestion using enzymes were subsequently isolated and quantified. The final step was to store all recovered products at −20°C.

The recombination reaction was carried out in accordance with instructions provided by the ClonExpress^®^ II one-step cloning kit. After that, Trelief^TM^5α chemically competent cells (Tsingke, Beijing, China) were developed by using the recombinant product. Selected individual colonies were transferred into tubes containing 15 mL of ampicillin-resistant liquid broth medium, and the tubes were agitated for 16 h. After assessing the size of the plasmid via double-restriction endonuclease digestion, the plasmid was purified from 10 mL of bacterial culture using the EndoFree Mini Plasmid Kit II (Tsingke, Beijing, China). The vector, meticulously designed as PcDNA3.1 (+)-CRABP1, was stored at a temperature of −20 °C.

### 2.5. CRABP1 Small Interfering RNA (siRNA) Synthesis

China’s Shanghai GenePharma Co., Ltd. (Shanghai, China) designed and developed the CRABP1 siRNA. The sequences are presented in Table 2.

### 2.6. Cell Transfection

In 6-well plates, the DPCs with excellent growth status were properly injected. After achieving a cell confluence of 50–80%, we employed the jetPRIME^®^ transfection reagent (Polyplus transfection, Illkirch, France) for the transfection of DNA or siRNA into dermal papilla cells (DPCs).

### 2.7. Cell Cycle Assay

The Cell Cycle and Apoptosis Analysis Kit (Beyotime, Shanghai, China) was utilized to evaluate the distribution of cells within the G1, S, and G2 phases that demonstrated alterations in dermal papilla cells (DPCs) of Hu sheep. We conducted this analysis 36 h after transfection once cell confluence reached 80%. The red fluorescence of more than 20,000 cells was quantified utilizing the BD LSRFortessa flow cytometer (BD, Franklin Lakes, NJ, USA). A wavelength of 488 nm activated these during the staining process. After flow cytometry, the data were evaluated using Modfit (Version: 3.1), and calculation was performed to measure the proportions of cells within the G1, S, and G2 phases.

### 2.8. Cell Counting Kit-8 Assay

In order to evaluate CCK-8, DPCs had been cultured on a 96-well cell culture plate. Cells were transfected once the DPC density reached 30%. The CCK-8 Kit’s instruction manual (Vazyme, Nanjing, China) was used to measure cell viability at 12, 24, 36, and 48 h after DPC transfection. Using a multi-mode micropore detection system, the absorbance at 450 nm was measured (Waltham, MA, USA; EnSpire, PerkinElmer).

### 2.9. Edu Assay

For the Edu experiment, dermal papilla cells (DPCs) were cultivated in a 24-well cell culture plate. Cells were transfected when the DPCs had achieved 50% density. After two days, we treated DPCs with a 4% paraformaldehyde treatment for a duration of 30 min. Subsequently, Triton X-100 was employed to render the membranes of cells permeable. During final stages of the experiment, DPCs were treated with the EdU Apollo at Vitro Imaging Kit (RiboBio, Guangzhou, China). Using a fluorescence-inverted microscope (DMi8, Leica, Germany), DPCs were stained, and images of the stained cellswere captured. The counting of stained cells in three randomly selected regions and the evaluation of the results was performed by using Image-Pro Plus 6.0 software from Media Cybernetics in Rockville, MD, USA.

### 2.10. Immunofluorescence Assay

In a 24-well plate, dermal papilla cells (DPCs) were cultivated for the immunofluorescence study. Once the cells reached approximately 50% confluence, transfection was performed following the standard cell transfection protocol. Cells designated for marker gene expression analysis were not subjected to transfection. Upon reaching the appropriate density, DPCs were rinsed with 1× PBSto prepare them for effective fixation. After that, the cells were fixed with 4% paraformaldehyde and permeabilized with 0.5% Triton X-100 (Solarbio in Beijing, China). After permeabilization, the cells were washed again with 1× PBS and blocked with 5% BSA (Solarbio, Beijing, China) at 37 °C. The cells were placed at 4 °C overnight in darkness after the treatment of the primary antibody. After a 12 h incubation in darkness at 37 °C, the cells were washed with 1× PBST (from Solarbio, Beijing, China), and was added the appropriate secondary antibody. Nuclear staining was performed using DAPI (Beyotime, Shanghai, China). A fluorescence-inverted microscope (Nikon, Tokyo, Japan) was employed for imaging to assess the staining. The primary antibodies used included anti-β-catenin (Beyotime, Shanghai, China) at a 1:400 dilution, and the secondary antibody used was Goat Anti-Mouse IgG H&L at a 1:400 dilution.

### 2.11. Data Analysis

A total of three independent experiments were performed for qPCR to ensure statistical significance and reproducibility. Each independent experiment was carried out with different cell cultures to account for biological variability. The data from the experiment were examined with Excel 2007 and SPSS 17.0. The GraphPad Prism 8 was used to generate the graphs. The data are shown as the mean with a standard error of the mean (SEM).The 2-delt delt-CT method was employed for the RT-PCR results. Independent sample *t*-tests and one-way ANOVA were used for the variance analysis. A *p*-value below 0.05 indicates a statistically significant difference, whereas a *p*-value less than 0.01 represents a highly significant difference.

## 3. Results

### 3.1. Overexpression and Inhibition of CRABP1 in the DPCs of Hu Sheep

**Figure 1:**
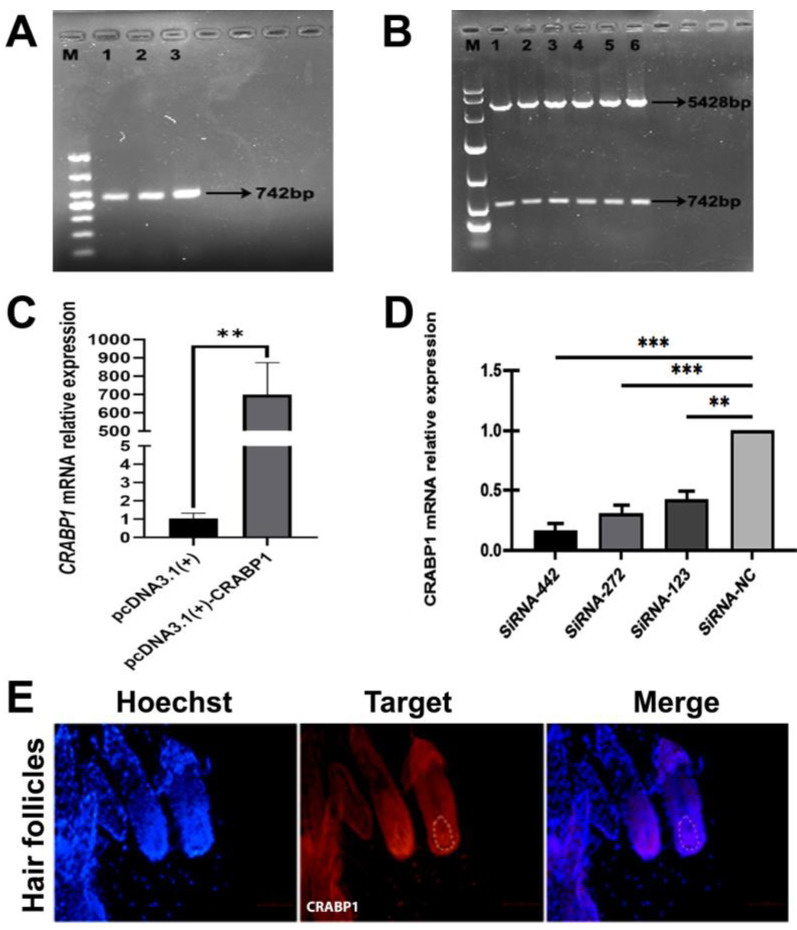
The construction of PcDNA3.1 (+)—CRABP1. (**A**) The CRABP1 coding sequence (CDS) of Hu sheep was amplified to its full length. Lanes 1, 2, and 3 are PCR products, while lane M is the DL 5000 marker. (**B**) Double enzymes digestion verification of PcDNA3.1 (+)-CRABP1. Lanes 1–6 represent the PCR product, and lane M is the DL10,000 marker. (**C**) The mRNA expression level in the DPCs of Hu sheep after CRABP1 transfection (*n* = 3 samples/group). (**D**) The relative expression levels of CRABP1 mRNA were assessed after transfection with different siRNA. (**E**) Immunofluorescence identification of CRABP1 in hair follicle of Hu sheep DPCs. Scale bar, 50 µm. (**) stands for highly significant differences (*p* < 0.01), and (***) stands for highly significant differences (*p* < 0.001).

According to the sequence provided by NCBI, the CDS region sequence of CRABP1 was successfully amplified. The PCR product’s gel electrophoresis revealed a strong band at 742 bp, as expected (Figure 1A). After constructing the overexpression vector (PcDNA3.1-CRABP1), we conducted a double enzyme digestion experiment. As a result, two bands were detected, as expected (Figure 1B), indicating the successful construction of PcDNA3.1-CRABP1 eukaryotic overexpression. Then, PcDNA3.1-CRABP1 was transfected to DPCs, and the mRNA expression level of CRABP1 was measured using RT-qPCR. The result revealed that mRNA expression levels of CRABP1 were significantly enhanced in the DPCs (*p* < 0.01) (Figure 1C) and proved that the CRABP1 overexpression vector could work well and could be used for further experiments.

To effectively inhibit CRABP1 in Hu sheep DPCs, the DPCs were transfected with different siRNA. RT-qPCR was used to measure the levels of CRABP1 mRNA expression (Figure 1D).These results support the idea that siRNA-442 could be used for further experiments.

Similarly, we used immunofluorescence to verify the expression pattern of CRABP1 in Hu sheep hair follicles, as shown in Figure 1E. The results indicated that CRABP1 is significantly expressed in the dermal papilla region, suggesting a potential role for CRABP1 in dermal papilla cells (DPCs).

### 3.2. CRABP1 Promotes DPCs Proliferation of Hu Sheep

**Figure 2:**
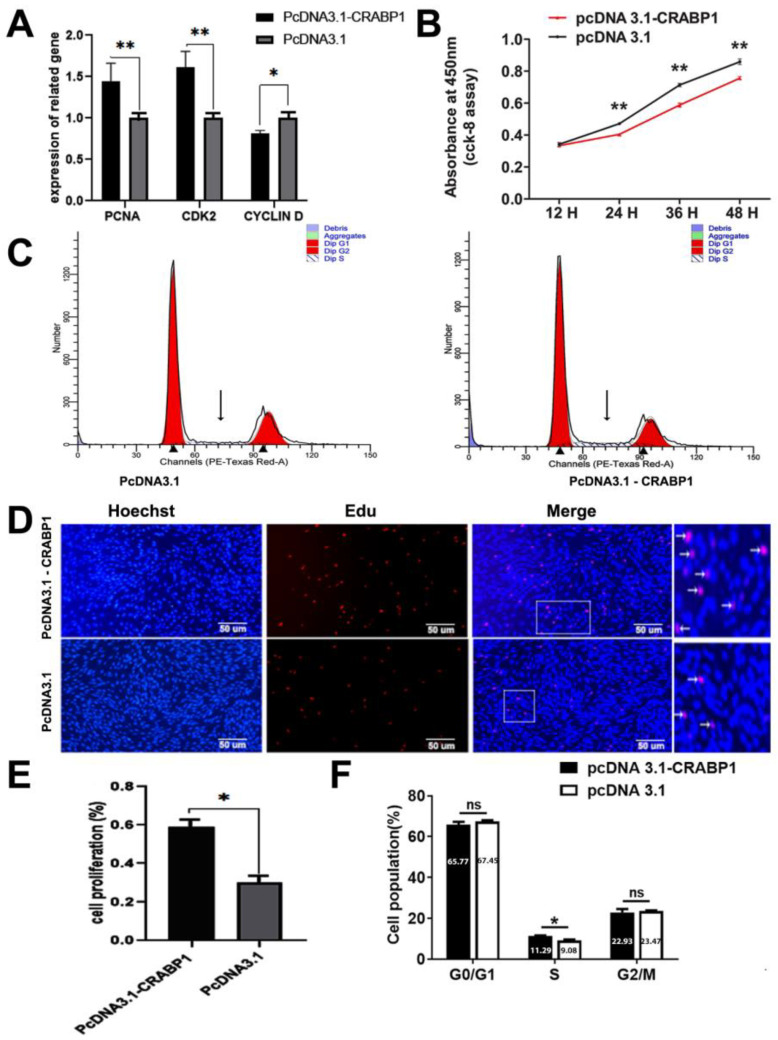
The influence of overexpressing CRABP1 on theDPCs proliferationin Hu sheep. (**A**) After overexpressing CRABP1, the mRNA levels of PCNA, CDK2, and CCND1 were measured. (**B**) CCK-8 is employed to evaluate DPC activity after the overexpression of the CRABP1 gene. (**C**) Flow cytometry is employed to analyze the cell cycle of DPC after CRABP1 overexpression. (**D**) The EDU test was applied to quantify the proliferation of DPCs in Hu sheep after CRABP1 overexpression. Scale bar, 50 µm. (**E**) The percentage of DPCs that test positive for EDU in Hu sheep. (**F**) The proportion of various phases within the cell cycle. (* *p* < 0.05, ** *p* < 0.01) denotes statistical significance. (ns) stands for highly significant differences (*p* > 0.05).

Subsequently, we studied the effect of overexpression CRABP1 on the proliferation of DPCs. After overexpression, RT-qPCR measured mRNA levels of related genes, including PCNA, CDK2, and CCND1.The findings indicate that the CRABP1 overexpression highly elevated the mRNA levels of PCNA and CDK2,while the *CCND1* mRNA expression level was found to have slightly decreased (Figure 2A). The CCK-8 assay revealed that the optical density (OD) values of the CRABP1 overexpression group were extensively greater than the control group at 24 and 48 h (*p* < 0.01) (Figure 2B), indicating an upregulation of cell viability. The EDU assay revealed that after the transfection of the pcDNA3.1-CRABP1 overexpression plasmid, the proportion of EDU-positive cells was markedly elevated in comparison to the control group. (*p* < 0.05) (Figure 2D,E). The cell cycle was examined by flow cytometry, which revealed that the percentage of S-phase cells increased significantly after transfection with PcDNA3.1CRABP1 in comparison to the control group (Figure 2C,F). The findings of CCK-8 and EDU assay indicate that the CRABP1 overexpression has a significant impact on DPC proliferation and viability.

**Figure 3:**
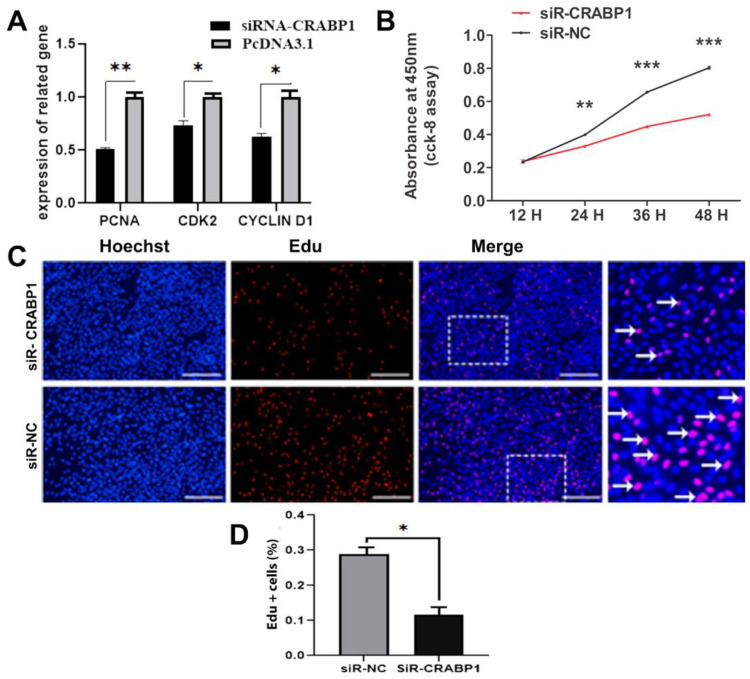
The effect of downregulation of CRABP1 on the DPCs proliferation in Hu sheep. (**A**) The comparative expression mRNA levels of PCNA, CDK2, and CCND1 after CRABP1 knockdown. (**B**) DPC activity is detected by CCK-8 after CRABP1 knockdown plasmid. (**C**) The EDU assay was used to measure the percentage of proliferating DPCs in Hu sheep after the downregulation of CRABP1. Proliferating cells are indicated by red EdUstaining, while nuclei are indicated by blue Hoechst staining. Scale bar, 50 µm. (**D**) The percentage of Hu sheep DPCs that are EdU-positive. * *p* < 0.05; ** *p* < 0.01; ***p < 0.001.

Furthermore, the CRABP1 knockdown plasmid was transfected into DPCs to verify the above results. After CRABP1 knockdown, there was a high decrease in the mRNA levels of *PCNA* (*p* < 0.01), *CCND1*, and *CDK2* (*p* < 0.05) (Figure 3A). The proliferation rate of DPCs, assessed using the CCK-8 assay, exhibited a significant decline at 24 to 48 h after transfection when compared to the control group (*p* < 0.001). At 48 h after transfection, this difference was highly significant (Figure 3B). According to the EDU assay, when the CRABP1 knockdown plasmid was introduced into DPCs, the proportion of cells that tested positive for EDU significantly declined compared to the control group (*p* < 0.05) (Figure 3C,D). These findings indicated that DPC proliferation could be inhibited by CRABP1 knockdown.

### 3.3. CRABP1 Regulates Nuclear Translocation of β-catenin

**Figure 4:**
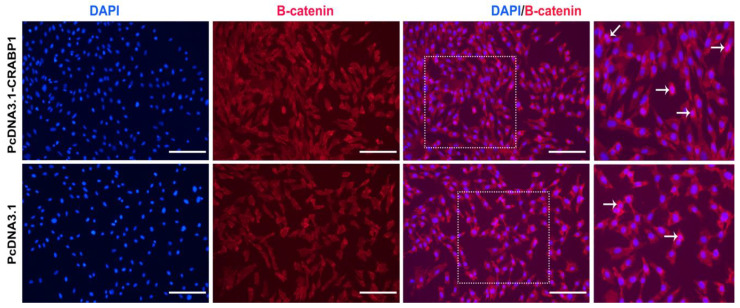
CRABP1 regulates nuclear translocation β-catenin. After CRABP1 overexpression, β-catenin was stained by immunofluorescence in DPCs. Red fluorescence showed the expression of β-catenin. Nucleus was stained with DAPI in blue. Scale bar, 50 µm.

To investigate how CRABP1 influences the movement of β-catenin into the cell nucleus, which is crucial for regulating gene expression. In Wnt signaling pathways, β-catenin moves to the nucleus to activate or repress target genes. We introduced CRABP1 overexpression into DPCs. The immunofluorescence staining revealed that the expression of β-catenin in the positive cells is higher as compared to the control group (Figure 4). The findings indicate that CRABP1 may regulate the nucleartranslocation of β-catenin in DPCs, which could facilitate processes such as cell differentiation and proliferation in DPCs of Hu sheep.

### 3.4. CRABP1 Regulated the Important Genes in the Wnt/β-catenin Signaling Pathway

**Figure 5:**
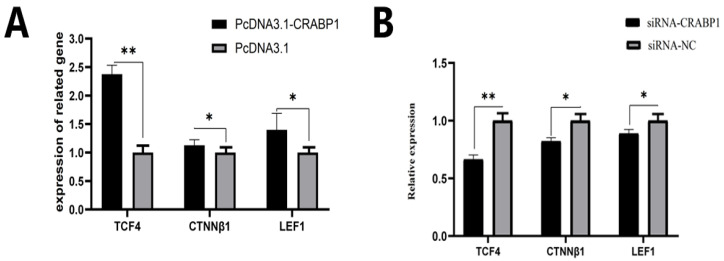
After CRABP1 transfection, both overexpression and knockdown of genes are observed in the Wnt/β-catenin signaling pathway. (**A**) The effect of CRABP1 overexpression on the gene expression of the Wnt/β-catenin signaling pathway. (**B**) The expression of genes in the Wnt/β-catenin signaling pathways is influenced by the downregulation of CRABP1. A “*” indicates a significant difference (*p* < 0.05), while a “**” indicates a highly significant difference (*p* < 0.01).

In light of the Wnt/β-catenin signaling pathway, it significantly influences dermal papilla cells (DPCs) and plays an indispensable role in regulating cell proliferation. We quantify the mRNA levels expression of important genes involved within this pathway (*CTNNβ1*, *TCF4*, and *LEF1*) after determining both CRABP1 overexpression and knockdown by RT-qPCR. The result indicated that the overexpression of CRABP1 significantly enhanced the extent of mRNA in *TCF4* (*p* < 0.01), *LEF1*, and *CTNNβ1* (*p* < 0.05) (Figure 5A). After the downregulation of CRABP1, the mRNA of *LEF1* and *CTNNβ1* were highly decreased (*p* < 0.05), although the expression levels of TCF4 mRNA were highly reduced (*p* < 0.01) (Figure 5B). The findings showed that CRABP1 regulates the expression of important genes associated with the Wnt/β-catenin signaling pathway in DPCs.

## 4. Discussion

Dermal papilla cells (DPCs) are specialized cells found at the base of hair follicles within the dermis layer of the skin. They are extremely important for regulating the cycle and growth of hair [27]. The initiation and regulation of the hair development cycle depend on DPCs [28]. During the anagen phase of the cycle, their proliferation is closely related to the initiation of new hair growth [29,30]. The growth of hair shafts and the development of hair follicles are significantly influenced by DPCs [31]. The dermal papilla (DPCs) originates from dermal condensate, which is formed by interactions between ectodermal cells and the formation of hair follicles during embryonic development [25,32]. The DPCs are the signaling core that regulates the proliferation and differentiation of matrix cells and stem cells derived from hair follicles, hence controlling the morphogenesis of hair follicles and cycles [33,34]. A number of genes, specifically CRABP1 [35], SOX2 [36], and FGF7/10 [37], have been associated with the hair growth cycle. All of these research findings indicate that dermal papilla cells (DPCs) are essential for hair follicle development. During the processes of developmental morphogenesis, adult hair follicle cycling, and skin tumor formation, CRABP1 and CRABP2 are dynamically regulated. Their distinct expression patterns in the dermis and epidermis align with their roles in the temporal and spatial regulation of RA signaling [38]. CRABP-I regulates retinoic acid (RA) metabolism by influencing the activity of RA-metabolizing enzymes. Increased CRABP-I expression in F9 teratocarcinoma cells enhances RA degradation and reduces sensitivity to RA-induced differentiation [8]. The F9 cells’ sensitivity to RA-induced differentiation inversely correlates with CRABP-I cellular level [17]. Moreover, CRABP-I modulates cellular responses to RA by promoting its catabolism or sequestering it from nuclear receptors. In contrast, CRABP-II interacts directly with retinoic acid receptors (RAR), facilitating RAR.RA complex formation. According to previous studies, CRABP2 is extensively expressed in hair follicles, whereas CRABP1 is exclusively expressed in the dermal papilla of healthy skin [39,40]. CRABP1 functions as a retinoic acid-binding protein and is found in the hair follicle DP of normal postnatal epidermis. This protein may have an effect on hair-bending regulators in mice [41]. Cellular retinoic acid-binding proteins (CRABPI) play an indispensable role in skin homeostasis and carcinogenesis, likely by regulating intracellular retinoid trafficking [41]. Nonetheless, the particular functions of CRABPs in sheep hair follicles have not been fully understood. Previous studies have demonstrated that dermal papilla cells express more CRABP1 than other hair follicle [42]. Thus, we concluded that CRABP1 may have an important effect on facilitating the development and proliferation of DPCs. We have demonstrated that CRABP1 overexpression can increase DPC proliferation in vitro based on RT-PCR, Immunostaining, CCK-8, EDU, and flow cytometry measurements.

The primary mechanism regulating DPC development is the Wnt/β-catenin signaling system, which mediates cell-to-cell signal transmission and plays a role in differentiation, apoptosis, and anti-apoptosis [43]. Moreover, the Wnt/β-catenin pathway is essential for the DP to maintain its ability to promote hair development and the proliferation of hair papilla cells [32,44]. According to the previous study results, we investigated the influence of the Wnt/β-catenin signaling pathway on the dermal papilla cells (DPCs) of Hu sheep. It has been suggested that the Wnt/β-catenin signaling pathway, which promotes the formation of human DPCs, may have a similar effect on Hu sheep DPCs [45]. In addition, the increase in the number of DPCs could potentially be reduced by inhibiting the Wnt/β-catenin pathway. Consequently, ICG001 was finally selected and integrated into DPCs because of its demonstrated ability to suppress the activation of the Wnt/β-catenin pathway [46]. Subsequently, it was observed that upregulation of CRABP1 could restore its functionality in the presence of Wnt/β-catenin pathway suppression. Furthermore, the proliferative capacity of DPCs shows partial recovery after the upregulation of CRABP1. Collectively, these findings show that CRABP1 stimulates the Wnt/catenin pathway, which promotes the development of DPCs. Previous research has shown that the Wnt/β-catenin signaling pathway plays a crucial role in the early development of hair follicles. Research has identified a relationship between the expression of CRABP1 and CRABP2 and the Wnt/β-catenin signaling pathway in mouse hair follicles [32,45]. Nevertheless, the impact of CRABP1 across the Wnt/β-catenin signaling pathway in DPCs from Hu sheep remained uncertain. Furthermore, CTNNB1, a downstream effector in the Wnt/β-catenin signaling pathway, is associated with the process of stem cell growth and the ability to regenerate themselves [45]. It has been found that TCF4 and LEF1, two other downstream effectors of the Wnt pathway, interact with CTNNB1 to regulate the expression of the target gene [4,47]. To explore, we overexpressed and knocked downCRABP1 to determine its potential role in regulating the Wnt/β-catenin signaling pathway and its effects on the biological functions of dermal papilla cells (DPCs).We measured the mRNA expression levels of TCF4, LEF1, CTNNβ1, and β-catenin. These findings indicate that CRABP1 has the potential to regulate the Wnt/β-catenin signaling pathway.

## 5. Conclusions

Our investigation suggests that CRABP1 may promote proliferation by the Wnt/β-catenin signaling pathway in the DPCs of Hu sheep. This study offers a basis for exploring molecular mechanisms regulating the growth of hair follicles.

## Figures and Tables

**Table 1:** Information on the primers.

Gene	Forward. Seq (5′-3′)	Reverse. Seq (5′-3′)	Product Size (bp)
CRABP1	ATGCCCAACTTCGCCGGC	TCATTCCCGAACATAAATCCTCG	94
PCNA	CGAGGGCTTCGACACTTAC	GTCTTCATTGCCAGCACATT	97
CDK2	AGAAGTGGCTGCATCACAAG	TCTCAGAATCTCCAGGGAATAG	92
CCND1	CCGAGGAGAACAAGCAGATC	GAGGGTGGGTTGGAAATG	91
GAPDH	TCTCAAGGGCATTCTAGGCTAC	GCCGAATTCATTGTCGTACCAG	151
CTNNB1	GAGGACAAGCCACAGGATTAT	CCAAGATCAGCGGTCTCATT	101
TCF4	CACTTTCCCTAGCTCCTTCTTC	GTAGCTGCTAGACTGTGGAATG	136
LEF1	CAGGTGGTGTTGGACAGATAA	ATGAGGGATGCCAGTTGTG	170

**Table 2:** The sequence of CRABP1 siRNA and NC.

Name	Forward Primer (5′-3′)	Reverse Primer (5′-3′)
SiRNA-442	GACGAACUCCUGACGUTT	ACGUCAGGAGUUCGUCTT
SiRNA-123	GCGCAGCAGCGAGAAUUUCTT	GAAAUUCUCGCUGCUGCGCTT
SiRNA-272	GCACGACCGAGAUCAACUUTT	AAGUUGAUCUCGGUCGUGCTT
SiRNA-NC	UUCUCCGAACGUGUCACGUTT	ACGUGACACGUUCGGAGAATT

## Data Availability

The raw data supporting the conclusions of the article will be made available by the authors without undue reservation.
